# Di-μ-oxido-bis­[(2-eth­oxy-6-{[2-(2-hy­droxy­ethyl­amino)­ethyl­imino]­meth­yl}phenolato-κ^3^ *N*,*N*′,*O* ^1^)oxidovanadium(V)]

**DOI:** 10.1107/S160053681105094X

**Published:** 2011-12-10

**Authors:** Fu-Ming Wang

**Affiliations:** a Key Laboratory of Coordination Chemistry and Functional Materials in Universities of Shandong, Dezhou University, Dezhou Shandong 253023, People’s Republic of China

## Abstract

In the title centrosymmetric dinuclear dioxidovanadium(V) complex, [V_2_(C_13_H_19_N_2_O_3_)_2_O_4_], the V^V^ ion is coordinated by an *N*,*N*′,*O*-tridendate 2-eth­oxy-6-{[2-(2-hy­droxy­ethyl­amino)­ethyl­imino]­meth­yl}phenolate ligand and three oxide O atoms, forming a distorted *cis*-VN_2_O_4_ octa­hedral geometry. The bridging O atoms show one short and one long bond to their two attached V^V^ atoms. The dihedral angle between the benzene ring of the ligand and the V_2_O_2_ plane is 75.2 (3)°. The deviation of the V^V^ ion from the plane defined by the three donor atoms of the tridentate ligand and one bridging oxide O atom is 0.337 (2) Å towards the terminal oxide O atom. Two N—H⋯O hydrogen bonds help to establish the conformation of the dimer. In the crystal, the complex mol­ecules are linked by O—H⋯O hydrogen bonds, forming [100] chains.

## Related literature

For background to vanadium complexes with Schiff base ligands, see: Kwiatkowski *et al.* (2006 ▶); Mondal *et al.* (2007 ▶); Rayati *et al.* (2007 ▶, 2008 ▶); Mikuriya & Matsunami (2005 ▶).
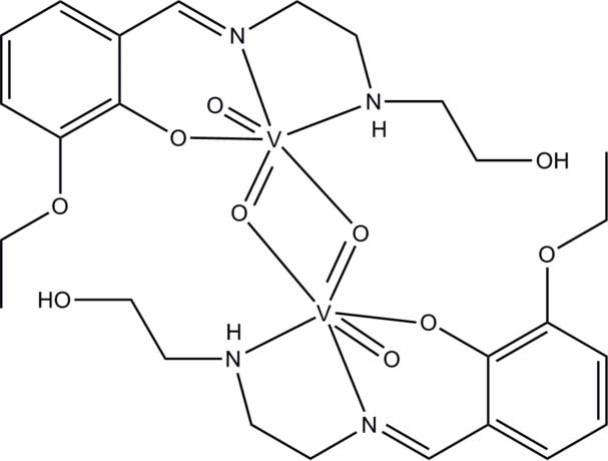


## Experimental

### 

#### Crystal data

[V_2_(C_13_H_19_N_2_O_3_)_2_O_4_]
*M* *_r_* = 668.48
Monoclinic, 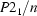

*a* = 9.907 (3) Å
*b* = 6.793 (2) Å
*c* = 22.279 (3) Å
β = 94.886 (2)°
*V* = 1493.9 (7) Å^3^
*Z* = 2
Mo *K*α radiation
μ = 0.69 mm^−1^
*T* = 298 K
0.20 × 0.18 × 0.17 mm

#### Data collection

Bruker SMART CCD diffractometer
Absorption correction: multi-scan (*SADABS*; Sheldrick, 1996 ▶) *T*_min_ = 0.875, *T*_max_ = 0.892
11652 measured reflections
3246 independent reflections
2485 reflections with *I* > 2σ(*I*)
*R*_int_ = 0.042

#### Refinement

*R*[*F* ^2^ > 2σ(*F* ^2^)] = 0.055
*wR*(*F* ^2^) = 0.172
*S* = 1.05
3246 reflections
195 parameters
1 restraint
H atoms treated by a mixture of independent and constrained refinement
Δρ_max_ = 1.85 e Å^−3^
Δρ_min_ = −0.54 e Å^−3^

### 

Data collection: *SMART* (Bruker, 1998 ▶); cell refinement: *SAINT* (Bruker, 1998 ▶); data reduction: *SAINT*; program(s) used to solve structure: *SHELXS97* (Sheldrick, 2008 ▶); program(s) used to refine structure: *SHELXL97* (Sheldrick, 2008 ▶); molecular graphics: *SHELXTL* (Sheldrick, 2008 ▶); software used to prepare material for publication: *SHELXTL*.

## Supplementary Material

Crystal structure: contains datablock(s) global, I. DOI: 10.1107/S160053681105094X/hb6538sup1.cif

Structure factors: contains datablock(s) I. DOI: 10.1107/S160053681105094X/hb6538Isup2.hkl

Additional supplementary materials:  crystallographic information; 3D view; checkCIF report

## Figures and Tables

**Table 1:** Selected bond lengths (Å)

V1—O5	1.634 (2)
V1—O4^i^	1.678 (2)
V1—O1	1.918 (2)
V1—N1	2.149 (3)
V1—N2	2.188 (3)
V1—O4	2.351 (2)

Symmetry code: (i) 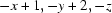.

**Table 2:** Hydrogen-bond geometry (Å, °)

*D*—H⋯*A*	*D*—H	H⋯*A*	*D*⋯*A*	*D*—H⋯*A*
N2—H2⋯O1^i^	0.90 (1)	2.20 (3)	3.033 (4)	154 (5)
O3—H3⋯O5^ii^	0.82	2.00	2.793 (4)	164

Symmetry codes: (i) 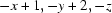; (ii) 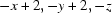.

**Fig. 1.**
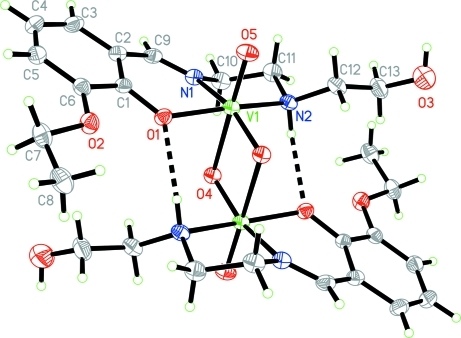
The molecular structure of the title complex with displacement ellipsoids are drawn at the 30% probability level. Unlabeled atoms are at the symmetry position (1-x, 2-y, -z).

**Fig. 2.**
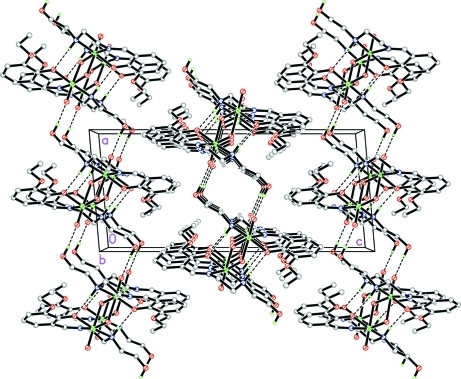
Molecular packing of the title complex, viewed along the b axis. Hydrogen bonds are shown as dashed lines.

## supplementary crystallographic information

### Comment

Schiff base compounds and their oxovanadium complexes have received much attention due to their structures and biological properties (Kwiatkowski *et al.*, 2006; Mondal *et al.*, 2007; Rayati *et al.*, 2008; Rayati *et al.*, 2007; Mikuriya & Matsunami, 2005). In this paper, the crystal structure of the title compound, (I), is reported.

The title complex is a centrosymmetric dinuclear dioxovanadium(V) compound, Fig. 1. The inversion center lies in the midpoint of the two V atoms. The V^V^ ion is coordinated by the phenolic O, imine N, and amine N atoms of a tridendate Schiff base ligand, and three oxo O atoms, forming a distorted octahedral geometry. The dihedral angle between the benzene ring and the V_2_O_2_ plane is 75.2 (3)°. The deviation of the V^V^ ion from the plane defined by the three donor atoms of the tridentate ligand and one bridging oxo O atom towards the terminal oxo O atom is 0.337 (2) Å. The coordinate bond lengths (Table 1) are comparable with those observed in similar oxovanadium(V) complexes cited above.

In the crystal, the complex molecules are linked through intermolecular O—H···O hydrogen bonds (Table 2), to form chains along the *a* axis (Fig. 2).

### Experimental

2-Hydroxy-3-ethoxybenzaldehyde (1 mmol, 0.17 g), 2-(2-aminoethylamino)ethanol (1 mmol, 0.10 g), and VO(acac)_2_ (1 mmol, 0.26 g) were mixed in methanol (30 ml). The mixture was boiled under reflux for 2 h, then cooled to room temperature. Brown blocks were formed after slow evaporation of the solution in air for a few days.

### Refinement

H2 atom was located from a difference Fourier map and refined isotropically. The N2—H2 distance is restrained to 0.90 (1) Å. The remaining hydrogen atoms were placed in geometrically idealized positions and constrained to ride on their parent atoms, with C—H distances of 0.93–0.97 Å, O—H distances of 0.82 Å, and with *U*_iso_(H) set at 1.2*U*_eq_(C) and 1.5*U*_eq_(C_methyl_ and O).

### Crystal data

**Table:**

[V_2_(C_13_H_19_N_2_O_3_)_2_O_4_]	*F*(000) = 696
*M**_r_* = 668.48	*D*_x_ = 1.486 Mg m^−^^3^
Monoclinic, *P*2_1_/*n*	Mo *K*α radiation, λ = 0.71073 Å
Hall symbol: -P 2yn	Cell parameters from 2450 reflections
*a* = 9.907 (3) Å	θ = 2.2–24.3°
*b* = 6.793 (2) Å	µ = 0.69 mm^−^^1^
*c* = 22.279 (3) Å	*T* = 298 K
β = 94.886 (2)°	Block, brown
*V* = 1493.9 (7) Å^3^	0.20 × 0.18 × 0.17 mm
*Z* = 2	

### Data collection

**Table:**

Bruker SMART CCD diffractometer	3246 independent reflections
Radiation source: fine-focus sealed tube	2485 reflections with *I* > 2σ(*I*)
graphite	*R*_int_ = 0.042
ω scan	θ_max_ = 27.0°, θ_min_ = 2.2°
Absorption correction: multi-scan (*SADABS*; Sheldrick, 1996)	*h* = −12→12
*T*_min_ = 0.875, *T*_max_ = 0.892	*k* = −8→8
11652 measured reflections	*l* = −28→26

### Refinement

**Table:**

Refinement on *F*^2^	Primary atom site location: structure-invariant direct methods
Least-squares matrix: full	Secondary atom site location: difference Fourier map
*R*[*F*^2^ > 2σ(*F*^2^)] = 0.055	Hydrogen site location: inferred from neighbouring sites
*wR*(*F*^2^) = 0.172	H atoms treated by a mixture of independent and constrained refinement
*S* = 1.05	*w* = 1/[σ^2^(*F*_o_^2^) + (0.0952*P*)^2^ + 1.1945*P*] where *P* = (*F*_o_^2^ + 2*F*_c_^2^)/3
3246 reflections	(Δ/σ)_max_ < 0.001
195 parameters	Δρ_max_ = 1.85 e Å^−^^3^
1 restraint	Δρ_min_ = −0.54 e Å^−^^3^

### Special details

**Table:**

Geometry. All esds (except the esd in the dihedral angle between two l.s. planes) are estimated using the full covariance matrix. The cell esds are taken into account individually in the estimation of esds in distances, angles and torsion angles; correlations between esds in cell parameters are only used when they are defined by crystal symmetry. An approximate (isotropic) treatment of cell esds is used for estimating esds involving l.s. planes.
Refinement. Refinement of F^2^ against ALL reflections. The weighted R-factor wR and goodness of fit S are based on F^2^, conventional R-factors R are based on F, with F set to zero for negative F^2^. The threshold expression of F^2^ > 2sigma(F^2^) is used only for calculating R-factors(gt) etc. and is not relevant to the choice of reflections for refinement. R-factors based on F^2^ are statistically about twice as large as those based on F, and R- factors based on ALL data will be even larger.

### Fractional atomic coordinates and isotropic or equivalent isotropic displacement parameters (Å^2^)

**Table:**

	*x*	*y*	*z*	*U*_iso_*/*U*_eq_	
V1	0.62902 (5)	0.97199 (8)	0.04516 (3)	0.0309 (2)	
N1	0.6461 (3)	1.2263 (4)	0.10267 (13)	0.0347 (6)	
N2	0.7184 (3)	1.1915 (4)	−0.01144 (13)	0.0342 (6)	
O1	0.5187 (2)	0.8713 (3)	0.10484 (10)	0.0351 (5)	
O2	0.4234 (3)	0.5847 (4)	0.16733 (11)	0.0424 (6)	
O3	0.9657 (4)	1.1068 (7)	−0.12850 (17)	0.0885 (12)	
H3	1.0385	1.0881	−0.1090	0.133*	
O4	0.4359 (2)	1.1593 (3)	0.01421 (10)	0.0351 (5)	
O5	0.7765 (2)	0.8771 (4)	0.06738 (12)	0.0432 (6)	
C1	0.5369 (3)	0.8873 (5)	0.16463 (14)	0.0329 (7)	
C2	0.5987 (3)	1.0530 (5)	0.19418 (16)	0.0368 (8)	
C3	0.6073 (4)	1.0645 (6)	0.25808 (17)	0.0456 (9)	
H3A	0.6472	1.1735	0.2777	0.055*	
C4	0.5572 (4)	0.9157 (7)	0.29065 (17)	0.0504 (10)	
H4	0.5622	0.9257	0.3324	0.060*	
C5	0.4984 (4)	0.7486 (6)	0.26289 (16)	0.0436 (9)	
H5	0.4681	0.6463	0.2861	0.052*	
C6	0.4852 (3)	0.7358 (5)	0.19995 (16)	0.0364 (8)	
C7	0.3486 (4)	0.4418 (6)	0.19921 (19)	0.0488 (10)	
H7A	0.2764	0.5054	0.2187	0.059*	
H7B	0.4081	0.3757	0.2297	0.059*	
C8	0.2907 (4)	0.2954 (6)	0.1524 (2)	0.0548 (11)	
H8A	0.2139	0.3527	0.1297	0.082*	
H8B	0.2630	0.1784	0.1722	0.082*	
H8C	0.3586	0.2625	0.1258	0.082*	
C9	0.6390 (3)	1.2243 (5)	0.16017 (16)	0.0372 (8)	
H9	0.6610	1.3394	0.1814	0.045*	
C10	0.6735 (4)	1.4124 (5)	0.07187 (18)	0.0425 (9)	
H10A	0.7142	1.5073	0.1005	0.051*	
H10B	0.5899	1.4676	0.0532	0.051*	
C11	0.7699 (4)	1.3655 (6)	0.02438 (17)	0.0459 (9)	
H11A	0.7769	1.4780	−0.0020	0.055*	
H11B	0.8594	1.3375	0.0436	0.055*	
C12	0.8244 (4)	1.0959 (6)	−0.04668 (18)	0.0435 (9)	
H12A	0.8988	1.0540	−0.0184	0.052*	
H12B	0.7852	0.9785	−0.0659	0.052*	
C13	0.8814 (4)	1.2206 (7)	−0.0943 (2)	0.0576 (11)	
H13A	0.8079	1.2757	−0.1206	0.069*	
H13B	0.9330	1.3286	−0.0754	0.069*	
H2	0.646 (3)	1.215 (8)	−0.0374 (18)	0.080*	

### Atomic displacement parameters (Å^2^)

**Table:**

	*U*^11^	*U*^22^	*U*^33^	*U*^12^	*U*^13^	*U*^23^
V1	0.0304 (3)	0.0253 (3)	0.0364 (4)	−0.0012 (2)	−0.0004 (2)	−0.0007 (2)
N1	0.0345 (14)	0.0279 (14)	0.0410 (16)	−0.0009 (11)	−0.0006 (12)	0.0005 (12)
N2	0.0301 (14)	0.0284 (15)	0.0437 (17)	−0.0034 (11)	0.0016 (12)	−0.0007 (12)
O1	0.0376 (12)	0.0345 (13)	0.0326 (12)	−0.0052 (10)	−0.0006 (10)	0.0003 (10)
O2	0.0471 (14)	0.0381 (14)	0.0427 (14)	−0.0078 (11)	0.0080 (11)	0.0048 (11)
O3	0.064 (2)	0.126 (4)	0.077 (2)	−0.002 (2)	0.0099 (18)	−0.019 (3)
O4	0.0361 (12)	0.0300 (12)	0.0390 (13)	−0.0020 (10)	0.0019 (10)	−0.0017 (10)
O5	0.0346 (13)	0.0363 (14)	0.0572 (16)	0.0015 (10)	−0.0039 (11)	0.0035 (12)
C1	0.0283 (16)	0.0367 (18)	0.0330 (17)	0.0040 (13)	−0.0011 (13)	0.0007 (14)
C2	0.0327 (17)	0.0387 (19)	0.0380 (19)	0.0019 (14)	−0.0029 (14)	−0.0006 (15)
C3	0.044 (2)	0.053 (2)	0.039 (2)	0.0049 (18)	−0.0068 (16)	−0.0089 (17)
C4	0.048 (2)	0.070 (3)	0.033 (2)	0.004 (2)	−0.0021 (16)	0.0011 (19)
C5	0.0390 (19)	0.050 (2)	0.042 (2)	0.0036 (17)	0.0042 (15)	0.0068 (17)
C6	0.0311 (17)	0.0367 (18)	0.0417 (19)	0.0040 (14)	0.0050 (14)	0.0032 (15)
C7	0.049 (2)	0.041 (2)	0.059 (3)	−0.0024 (17)	0.0194 (19)	0.0069 (18)
C8	0.052 (2)	0.042 (2)	0.072 (3)	−0.0074 (18)	0.018 (2)	0.002 (2)
C9	0.0330 (17)	0.0333 (18)	0.044 (2)	−0.0001 (14)	−0.0042 (14)	−0.0083 (15)
C10	0.045 (2)	0.0276 (18)	0.054 (2)	−0.0045 (15)	0.0008 (17)	−0.0031 (16)
C11	0.048 (2)	0.035 (2)	0.055 (2)	−0.0168 (16)	0.0055 (17)	−0.0032 (17)
C12	0.0335 (17)	0.042 (2)	0.056 (2)	0.0006 (15)	0.0055 (16)	−0.0030 (18)
C13	0.044 (2)	0.070 (3)	0.061 (3)	0.005 (2)	0.0186 (19)	0.010 (2)

### Geometric parameters (Å, °)

**Table:**

V1—O5	1.634 (2)	C3—H3A	0.9300
V1—O4^i^	1.678 (2)	C4—C5	1.396 (6)
V1—O1	1.918 (2)	C4—H4	0.9300
V1—N1	2.149 (3)	C5—C6	1.400 (5)
V1—N2	2.188 (3)	C5—H5	0.9300
V1—O4	2.351 (2)	C7—C8	1.518 (6)
N1—C9	1.289 (4)	C7—H7A	0.9700
N1—C10	1.475 (4)	C7—H7B	0.9700
N2—C11	1.492 (4)	C8—H8A	0.9600
N2—C12	1.510 (4)	C8—H8B	0.9600
N2—H2	0.895 (10)	C8—H8C	0.9600
O1—C1	1.333 (4)	C9—H9	0.9300
O2—C6	1.371 (4)	C10—C11	1.518 (5)
O2—C7	1.444 (4)	C10—H10A	0.9700
O3—C13	1.408 (5)	C10—H10B	0.9700
O3—H3	0.8200	C11—H11A	0.9700
O4—V1^i^	1.678 (2)	C11—H11B	0.9700
C1—C2	1.417 (5)	C12—C13	1.505 (5)
C1—C6	1.417 (5)	C12—H12A	0.9700
C2—C3	1.421 (5)	C12—H12B	0.9700
C2—C9	1.463 (5)	C13—H13A	0.9700
C3—C4	1.363 (6)	C13—H13B	0.9700
			
O5—V1—O4^i^	107.58 (12)	O2—C6—C5	125.2 (3)
O5—V1—O1	101.35 (12)	O2—C6—C1	114.5 (3)
O4^i^—V1—O1	98.86 (10)	C5—C6—C1	120.3 (3)
O5—V1—N1	96.52 (12)	O2—C7—C8	106.4 (3)
O4^i^—V1—N1	154.55 (11)	O2—C7—H7A	110.4
O1—V1—N1	83.89 (11)	C8—C7—H7A	110.4
O5—V1—N2	92.77 (12)	O2—C7—H7B	110.4
O4^i^—V1—N2	93.15 (11)	C8—C7—H7B	110.4
O1—V1—N2	157.67 (11)	H7A—C7—H7B	108.6
N1—V1—N2	77.33 (11)	C7—C8—H8A	109.5
O5—V1—O4	170.41 (10)	C7—C8—H8B	109.5
O4^i^—V1—O4	78.97 (11)	H8A—C8—H8B	109.5
O1—V1—O4	84.19 (9)	C7—C8—H8C	109.5
N1—V1—O4	76.14 (9)	H8A—C8—H8C	109.5
N2—V1—O4	79.70 (9)	H8B—C8—H8C	109.5
C9—N1—C10	119.9 (3)	N1—C9—C2	124.3 (3)
C9—N1—V1	125.2 (2)	N1—C9—H9	117.9
C10—N1—V1	114.8 (2)	C2—C9—H9	117.9
C11—N2—C12	113.4 (3)	N1—C10—C11	107.2 (3)
C11—N2—V1	111.6 (2)	N1—C10—H10A	110.3
C12—N2—V1	109.9 (2)	C11—C10—H10A	110.3
C11—N2—H2	115 (3)	N1—C10—H10B	110.3
C12—N2—H2	107 (3)	C11—C10—H10B	110.3
V1—N2—H2	99 (3)	H10A—C10—H10B	108.5
C1—O1—V1	128.8 (2)	N2—C11—C10	109.4 (3)
C6—O2—C7	117.9 (3)	N2—C11—H11A	109.8
C13—O3—H3	109.5	C10—C11—H11A	109.8
V1^i^—O4—V1	101.03 (11)	N2—C11—H11B	109.8
O1—C1—C2	123.0 (3)	C10—C11—H11B	109.8
O1—C1—C6	118.1 (3)	H11A—C11—H11B	108.2
C2—C1—C6	118.8 (3)	C13—C12—N2	116.3 (3)
C1—C2—C3	119.7 (3)	C13—C12—H12A	108.2
C1—C2—C9	121.2 (3)	N2—C12—H12A	108.2
C3—C2—C9	118.6 (3)	C13—C12—H12B	108.2
C4—C3—C2	119.9 (4)	N2—C12—H12B	108.2
C4—C3—H3A	120.0	H12A—C12—H12B	107.4
C2—C3—H3A	120.0	O3—C13—C12	110.3 (4)
C3—C4—C5	121.7 (4)	O3—C13—H13A	109.6
C3—C4—H4	119.2	C12—C13—H13A	109.6
C5—C4—H4	119.2	O3—C13—H13B	109.6
C4—C5—C6	119.6 (4)	C12—C13—H13B	109.6
C4—C5—H5	120.2	H13A—C13—H13B	108.1
C6—C5—H5	120.2		

Symmetry codes: (i) −*x*+1, −*y*+2, −*z*.

### Hydrogen-bond geometry (Å, °)

**Table:**

*D*—H···*A*	*D*—H	H···*A*	*D*···*A*	*D*—H···*A*
N2—H2···O1^i^	0.90 (1)	2.20 (3)	3.033 (4)	154 (5)
O3—H3···O5^ii^	0.82	2.00	2.793 (4)	164

Symmetry codes: (i) −*x*+1, −*y*+2, −*z*; (ii) −*x*+2, −*y*+2, −*z*.
